# Validation of a novel FRET real-time PCR assay for simultaneous quantitative detection and discrimination of human *Plasmodium* parasites

**DOI:** 10.1371/journal.pone.0252887

**Published:** 2021-06-04

**Authors:** Renate Schneider, Aline Lamien-Meda, Herbert Auer, Ursula Wiedermann-Schmidt, Peter L. Chiodini, Julia Walochnik

**Affiliations:** 1 Institute for Specific Prophylaxis and Tropical Medicine, Center for Pathophysiology, Infectiology and Immunology, Medical University of Vienna, Vienna, Austria; 2 UK NEQAS Parasitology, Public Health England, London, United Kingdom; University of Helsinki, FINLAND

## Abstract

Increasing numbers of travelers returning from endemic areas, migrants, and refugees have led to a significant rise in the number of imported malaria cases in non-endemic countries. Real- time PCR serves as an excellent diagnostic tool, especially in regions where experience in microscopy is limited. A novel fluorescence resonance energy transfer-based real-time PCR (FRET-qPCR) was developed and evaluated using 56 reference samples of the United Kingdom National External Quality Assessment Service (UK NEQAS) for molecular detection of malaria, including *P*. *falciparum*, *P*. *vivax*, *P*. *ovale*, *P*. *malariae*, and *P*. *knowlesi*. Species identification is based on single nucleotide polymorphisms (SNPs) within the genome where the MalLC640 probe binds, lowering the melting temperature in the melting curve analysis. The novel FRET-qPCR achieved 100% (n = 56) correct results, compared to 96.43% performing nested PCR. The high sensitivity, with a calculated limit of detection of 199.97 parasites/mL blood for *P*. *falciparum*, is a significant advantage, especially if low-level parasitemia has to be ruled out. Even mixed infections of *P*. *falciparum* with *P*. *vivax* or *P*. *ovale*, respectively, were detected. In contrast to many other real-time PCR protocols, this novel FRET-qPCR allows the quantitative and species-specific detection of *Plasmodium* spp. in one single run. Solely, *P*. *knowlesi* was detected but could not be differentiated from *P*. *vivax*. The turnaround time of this novel FRET-qPCR including DNA extraction is less than two hours, qualifying it for routine clinical applications, including treatment monitoring.

## Introduction

Malaria is still one of the most important infectious diseases worldwide and remains endemic in many low and middle income countries. Increasing numbers of travelers, migrants and refugees coming from different endemic areas have led to a significant rise in the number of imported malaria cases in non-endemic countries in recent years [1, 2]. Accurate diagnosis and treatment without delay are essential for reducing morbidity and mortality, especially in *P*. *falciparum* malaria.

The gold standard for malaria diagnostics still is the microscopic examination of Giemsa-stained thick and thin blood films. However, microscopy is time-consuming and requires considerable expertise, particularly at low-level parasitemia. Identifying the correct *Plasmodium* species can be demanding, especially in non-endemic countries, where qualified, experienced malaria microscopists are rare [2]. Moreover, the parasite´s morphology can be altered due to prophylactic or stand by medication [3]. Yet, correct species identification is essential since treatment depends on the infecting species [1, 2]. Rapid diagnostic tests (RDT) are highly convenient but not always fully reliable [3–6], thus molecular methods, particularly PCR-based methods have gained more and more importance in malaria diagnostics. Among numerous published PCR protocols, a nested PCR protocol still serves as the “molecular gold standard” and has been performed in our lab for many years [7]. However, real-time PCR protocols have the advantage of faster results, they are less labor-intensive, and the results are quantitative [3, 4, 8, 9]. Considering the apparent advantages of these methods, not all of the published *Plasmodium* real-time PCR protocols have been fully evaluated: e.g. some cannot differentiate between species [3, 10, 11], some do not detect all species [4, 5, 12] and for others, a genus-specific PCR has to be performed first and has to be followed by additional reactions [8]. These shortcomings are diminishing the apparent benefits of real-time PCR and are prolonging the time to obtain the final result. Hence the aim of the current study was to develop a novel real-time PCR assay that allows a rapid, highly sensitive, quantitative, and species-specific diagnosis of malaria and also to validate this PCR for routine diagnostics by evaluating its performance with international references samples.

## Material and methods

### Samples

A total of 56 lyophilized blood samples distributed by the United Kingdom National External Quality Assessment Service (UK NEQAS) for molecular detection of malaria between 2016 and 2020 were included in this study and investigated by the standard nested PCR [7] and a novel fluorescence resonance energy transfer-based real-time PCR (FRET-qPCR). UK NEQAS aims to improve the diagnosis of parasitic infections through monitoring laboratory performance, in an independent manner and on a non-for-profit basis. All UK NEQAS specimens were either from a single patient sample or from laboratory cultures (*P*. *falciparum* and *P*. *knowlesi*) and were diluted to the required parasite density using donor blood which was negative for malaria DNA.

The samples had already been tested by UK NEQAS with the standard nested PCR before and after their distribution.

### DNA extraction

As instructed by UK NEQAS, the lyophilized blood samples were reconstituted in 500 μL of molecular grade water. After 5 min. of incubation at room temperature, DNA was extracted from 200 μL of the samples with the QIAamp® DNA mini kit (Quiagen, Hilden, Germany) according to the manufacturer´s instructions.

### Nested PCR

The samples were analyzed blindly with the nested PCR protocol within three weeks of receipt and results were returned [7]. Additionally, samples that showed a positive reaction in the genus-specific nest2 reaction, but no reaction in any of the species-specific nest2 PCRs (*P*. *falciparum*, *P*. *vivax*, *P*. *ovale*, *P*. *malariae*), were also tested following published protocols for the detection of *P*. *knowlesi* [13, 14]. After all results from the participating laboratories (e.g. 89 laboratories in March 2020) had been processed, UK NEQAS provided data on the expected *Plasmodium* species and the number of parasites per mL blood. All *Plasmodium*-positive DNA samples (n = 46) and all remaining *Plasmodium*-negative samples (n = 10) were included in this study, stored at -20°C until further use.

### FRET-qPCR assay

**Fig 1:**
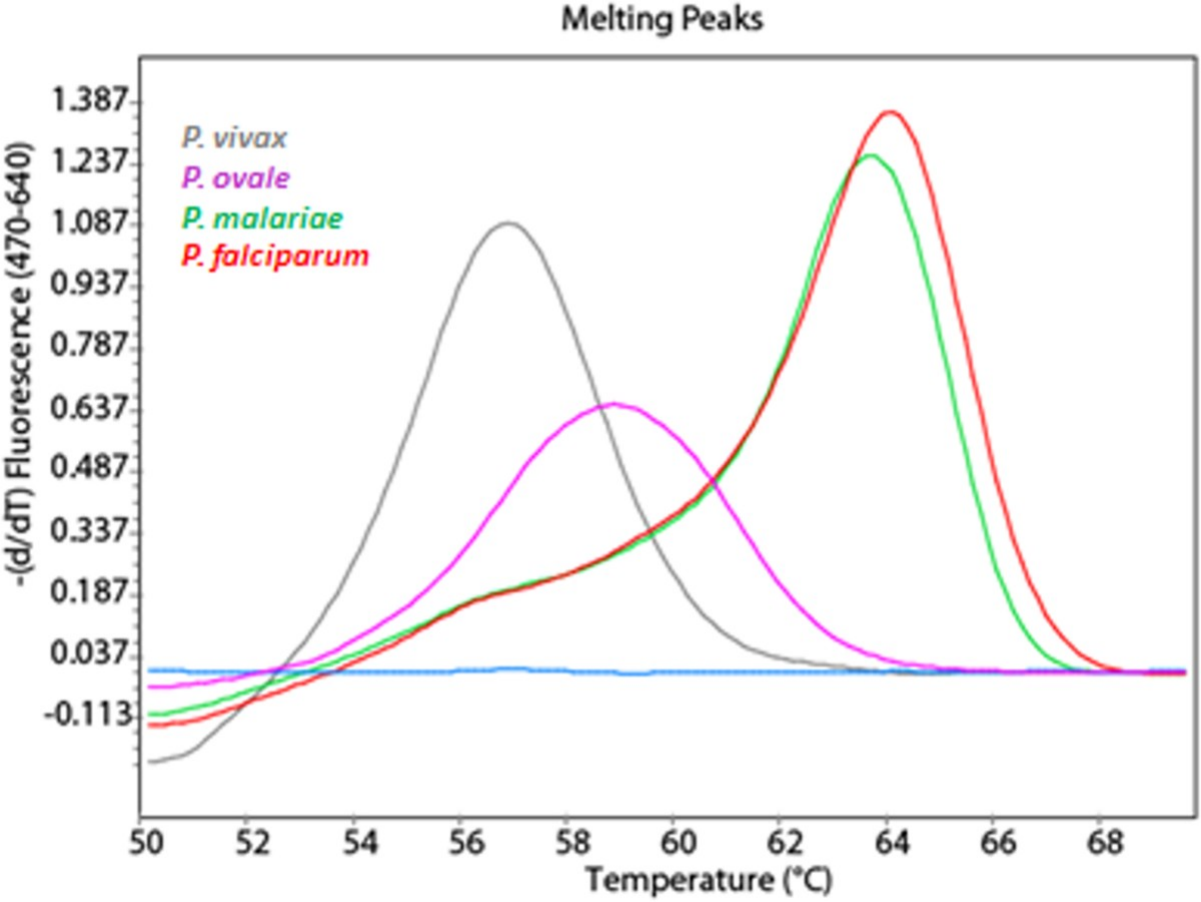
Species differentiation based on the Tm values. Melting curves of amplicons post real-time PCR from *P*. *falciparum* (red), *P*. *malariae* (green), *P*. *ovale* (lilac) and *P*. *vivax* (grey*)*.

The newly developed FRET-qPCR targets the small subunit 18S rRNA gene and amplifies 157–165 bp fragments, the length depending on the respective *Plasmodium* species. We used the primers Plasmo 1 (5´-GTTAAGGGAGTGAAGACGATCAGA-3´) and Plasmo 2 (5´-AACCCAAAGACTTTGATTTCTCATAA-3) [8] and designed two target-specific hybridization probes to allow fluorescence resonance energy transfer (FRET)-based detection of amplicons. The sequences of the hybridization probes MalFL (5´- CTTTCATCCAACACCTAGTCGGC; 3´label fluorescein) and MalLC640 (5´- TAGTTTATGGTTAAGATTACGACGGT; 5´label, LC red 640, 3´phosphorylated) are identical to *P*. *falciparum* (GenBank M19172, taxon ID: 5833). Fluorescence at 640 nm was generated by FRET following the annealing of both probes to their adjacent complementary sequences. Species discrimination was based on the presence of single nucleotide polymorphisms (SNPs), reducing the affinity of the MalLC640 probe for *P*. *vivax/ knowlesi (*2 mismatches*)*, *P*. *ovale* (1 mismatch*)*, and *P*. *malariae* (1 mismatch) and thus lowering the melting temperature (Tm) in the melting curve analysis (Fig 1). Tm is defined as the point at which half of the probes have melted off the DNA. Primers and probes were synthesized by TIB Molbiol GmbH (Berlin, Germany). PCR amplifications were performed in a reaction volume of 20 μL in sealed glass capillaries with a LightCycler 2.0 (Roche Diagnostics GmbH, Vienna, Austria), containing 1x LightCycler® FastStart DNA Master HybProbe kit, 4mM MgCl2, 4pmol of each hybridization probe, 20 pmol of each primer, and 5 μl of DNA template. In the FastStart DNA Master, HybProbe mix dTTP was replaced by dUTP, allowing the use of uracil-N-glycosylase (UNG) as an additional carryover prevention measure. Thermal cycling comprised an initial denaturation at 95°C for 10 min, followed by ten touchdown cycles (69–58°C) and 35 cycles of 95°C for 5 sec, 58°C for 10 sec, and 72°C for 15 sec. A final melting curve analysis was performed by initial denaturation at 95°C for 20 s, followed by 50°C for 20 s and continuous heating at 0.2°C/s to 70°C. Each run included negative and positive controls. Positive results were automatically determined by the software. The crossing point value (Cp) was inversely proportional to the initial *Plasmodium* sp. concentration in the sample and reflected only the cycles of isothermal annealing at 58°C. Melting curves and melting peaks were generated by the software and the resultant Tm values were calculated by the software. Only when the software failed to calculate the Tm properly, it was manually adjusted to peak maximum.

#### Specificity of the FRET-qPCR

In addition to the 46 *Plasmodium* positive and 10 *Plasmodium* negative samples listed in Table 1, the specificity of our novel PCR was evaluated with DNA from *Leishmania*, *Babesia*, *Pneumocystis*, and *Toxoplasma*.

**Table 1:** Performance of the reference samples in the standard nested PCR and in the novel FRET-qPCR.

Number	Year	UKNEQAS reported species	Parasite/mL	Nested PCR	FRET-qPCR species	Cp	Tm
uk3245	2016	*P*.*malariae*	5,000/mL	*P*.*malariae*	*P*.*malariae*	28	63.2
uk3246	2016	*P*.*ovale*	20,000/mL	*P*.*ovale curtisi*	*P*.*ovale*	22	58.2
uk3247	2016	*P*.*ovale*	5,000/mL	*P*.*ovale curtisi*	*P*.*ovale*	25	58.6
uk3372	2016	*P*.*vivax*	14,000,000/mL	*P*.*vivax*	*P*.*vivax/knowlesi*	23	56.7
uk3374	2016	*P*.*knowlesi*	10,000,000/mL	*P*.*knowlesi*	*P*.*vivax/knowlesi*	22	56.5
uk3375	2016	*P*.*falciparum*	10,000,000/mL	*P*.*falciparum*	*P*.*falciparum*	20	63.7
uk3504	2016	*P*.*vivax*	14,000,000/mL	*P*.*vivax*	*P*.*vivax/knowlesi*	20	56.5
uk3505	2016	*P*.*falciparum*	10,000,000/mL	*P*.*falciparum*	*P*.*falciparum*	19	63.7
uk3506	2016	*P*.*knowlesi*	10,000,000/mL	*P*.*knowlesi*	*P*.*vivax/knowlesi*	21	56.4
uk3635	2017	*P*.*ovale*	1,000/mL	*P*.*ovale curtisi*	*P*.*ovale*	26	59
uk3636	2017	*P*.*falciparum*	10,000,000/mL	*P*.*falciparum*	*P*.*falciparum*	20	63.6
**uk3637**	2017	***P*.*malariae***	**1,000/mL**	**0**	***P*.*malariae***	**>30**	**63.4**
uk3638	2017	*P*.*falciparum*	1,000,000/mL	*P*.*falciparum*	*P*.*falciparum*	27	63.7
uk3855	2017	*P*.*falciparum*	200/mL	*P*.*falciparum*	*P*.*falciparum*	>30	63.6
uk3856	2017	*P*.*knowlesi*	2,000/mL	*P*.*knowlesi*	*P*.*vivax/knowlesi*	26	56.8
uk3985	2017	0	0	0	0		
uk3986	2017	*P*.*knowlesi*	100,000/mL	*P*.*knowlesi*	*P*.*vivax/knowlesi*	20	56.3
uk3987	2017	*P*.*vivax*	10,000/mL	*P*.*vivax*	*P*.*vivax/knowlesi*	25	56.7
uk3988	2017	*P*.*falciparum*	5,000/mL	*P*.*falciparum*	*P*.*falciparum*	23	63.6
uk4123	2017	*P*.*falciparum*	20,000/mL	*P*.*falciparum*	*P*.*falciparum*	20	63.6
uk4125	2017	*P*.*knowlesi*	1,000/mL	*P*.*knowlesi*	*P*.*vivax/knowlesi*	24	56.5
uk4126	2017	*P*.*falciparum*	5,000/mL	*P*.*falciparum*	*P*.*falciparum*	23	63.7
uk4260	2018	0	0	0	0		
uk4261	2018	*P*.*knowlesi*	1,000/mL	*P*.*knowlesi*	*P*.*vivax/knowlesi*	28	57.1
uk4262	2018	*P*.*vivax*	1,000/mL	*P*.*vivax*	*P*.*vivax/knowlesi*	30	56.9
uk4463	2018	*P*.*falciparum*	200,000/mL	*P*.*falciparum*	*P*.*falciparum*	20	63.7
uk4464	2018	0	0	0	0		
uk4465	2018	*P*.*vivax*	46,400/mL	*P*.*vivax*	*P*.*vivax/knowlesi*	21	56.5
uk4466	2018	*P*.*ovale*	20,000/mL	*P*.*ovale curtisi*	*P*.*ovale*	24	58.6
uk4602	2018	*P*.*knowlesi*	20,000/mL	*P*.*knowlesi*	*P*.*vivax/knowlesi*	21	56.3
uk4603	2018	*P*.*falciparum*	20,000/mL	*P*.*falciparum*	*P*.*falciparum*	23	63.9
uk4605*	2018	*P*.*vivax*	1,000/mL	*P*.*vivax*	*P*.*vivax/knowlesi*	>30	57.1
uk4889	2019	*P*.*falciparum*	2,000,000/mL	*P*.*falciparum*	*P*.*falciparum*	14	63.7
uk4890	2019	*P*.*falciparum*	10,000/mL	*P*.*falciparum*	*P*.*falciparum*	23	63.6
uk4891	2019	*P*.*falciparum*	20,000,000/mL	*P*.*falciparum*	*P*.*falciparum*	15	63.7
uk4892	2019	*P*.*falciparum*	100,000/mL	*P*.*falciparum*	*P*.*falciparum*	20	63.7
uk5083	2019	*P*.*falciparum*	1,000,000/mL	*P*.*falciparum*	*P*.*falciparum*	16	63.8
uk5084	2019	*P*.*ovale*	65,000/mL	*P*.*ovale curtisi*	*P*.*ovale*	24	58.3
uk5085	2019	0	0	0	0		
uk5086	2019	*P*.*ovale*	20,000/mL	*P*.*ovale curtisi*	*P*.*ovale*	23	58.3
uk5219	2019	*P*.*knowlesi*	1,000,000/mL	*P*.*knowlesi*	*P*.*vivax/knowlesi*	14	56.5
uk5220	2019	0	0	0	0		
uk5221	2019	*P*.*falciparum*	10,000/mL	*P*.*falciparum*	*P*.*falciparum*	24	63.8
uk5222	2019	0	0	0	0		
uk5363	2019	0	0	0	0		
uk5364	2019	0	0	0	0		
uk5365	2019	*P*.*vivax*	6,400/mL	*P*.*vivax*	*P*.*vivax/knowlesi*	23	56.5
uk5366	2019	*P*.*vivax*	180/mL	*P*.*vivax*	*P*.*vivax/knowlesi*	25	56.7
uk5500	2020	*P*.*falciparum*	1,000,000/mL	*P*.*falciparum*	*P*.*falciparum*	16	63.9
uk5501	2020	*P*.*vivax*	12,000/mL	*P*.*vivax*	*P*.*vivax/knowlesi*	22	56.9
uk5502*	2020	***P*.*falciparum***	**50/mL**	***0***	***P*.*falciparum***	**>30**	**63.7**
uk5503	2020	0	0	0	0		
uk5848*	2020	*P*.*falciparum*	1,000/mL	*P*.*falciparum*	*P*.*falciparum*	30	63.7
uk5859	2020	*P*.*knowlesi*	218,000/mL	*P*.*knowlesi*	*P*.*vivax/knowlesi*	18	56.3
uk5850	2020	0	0	0	0		
uk5851	2020	*P*.*vivax*	15,000/mL	*P*.*vivax*	*P*.*vivax/knowlesi*	22	56.2

Cp: crossing point

*not scored: generated the correct result

**Bold**: false negative in the nested PCR

#### Sensitivity

Tenfold dilutions were prepared from a sample with 1,000,000 to 100 *P*. *falciparum*/mL blood (uk5083). To proof the limit of detection (LOD) additional dilutions were prepared with molecular grade water corresponding to 500, 200, 100, 50, and 25 *P*. *falciparum*/mL blood. Minimum 10 replicates were tested for each dilution and the results were analyzed by Statagraphics Version 18/19.

## Results

**Fig 2:**
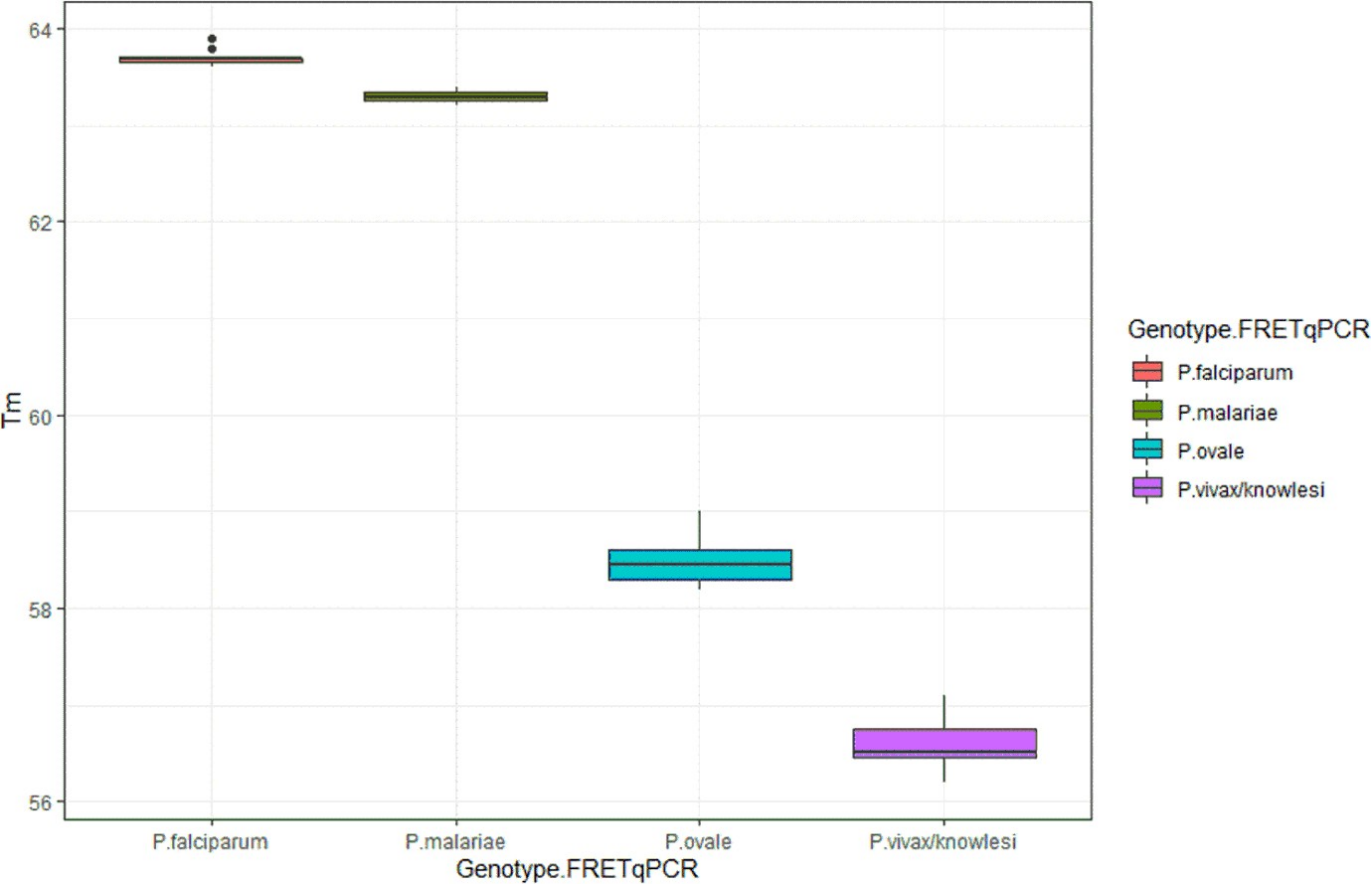
Boxplots of the melting temperature of *P*. *falciparum* (red), *P*. *malariae* (green), *P*. *ovale* (turquoise), *P*. *vivax/ knowlesi* (pink). The box specifies the likely range of melting temperature variation. The melting temperature ranges were 63.6–63.9 for *P*. *falciparum*, 63.2–63.4 for *P*. *malariae*, 58.2–59 for *P*. *ovale*, 56.2–57.1 for *P*. *vivax/ knowlesi*. The figure was generated using R version 3.4.2 (2017-09-28) via RStudio version Version 1.1.383.

Table 1 shows the results of the novel FRET-qPCR, comparing them to the expected result from the UK NEQAS report, and the results from the nested PCR performed in our lab between August 2016 and September 2020. According to the Tm value in the FRET-qPCR, the samples were assigned to *P*. *falciparum* (Tm 63.5–66°C), *P*. *malariae* (Tm 63.0–63.5°C), *P*. *ovale* (Tm 58–60°C), and *P*. *vivax/knowlesi* (Tm 56–57.5°C), respectively (Fig 1). The novel FRET-qPCR achieved 100% (n = 56) correct results compared to the nested PCR with 96.43%. Based on the determined Tm values, all positive samples were assigned to the correct *Plasmodium* species, except that *P*. *knowlesi* could not be differentiated from *P*. *vivax* because of their identical Tm value (S1 Fig). In total, 46 out of 56 samples were positive in the FRET-qPCR (19 *P*. *falciparum*, *19 P*. *vivax/knowlesi*, 6 *P*. *ovale*, 2 *P*. *malariae*), 10 were negative (Table 1, Fig 2).

The UK NEQAS samples comprised samples from 20,000,000 to 50 parasites/mL, showing Cp values between 14 and >30 (Table 1). It is important to note that samples from 2016 through 2018 underwent several freeze-thaw cycles until the FRET-qPCR was performed (as it was not established at that time), affecting the sensitivity (and thus the Cp values of the respective samples). Therefore, reliable statistical analysis of the correlation between the Cp values and parasite density was not possible. However, boxplots for the likely range of melting temperature variation for the different species are given in Fig 2.

### Specificity

The designation of the *Plasmodium* positive samples to the correct species on the basis of the Tm value in the last step of the FRET-qPCR is demonstrated in Fig 1. All *Plasmodium*-positive samples (n = 46) were designated to the correct species (Table 1, Fig 2). All available negative UK NEQAS samples (n = 10) and DNA samples from *Leishmania*, *Babesia*, *Pneumocystis*, *and Toxoplasma* showed no reaction in the FRET-qPCR.

### Sensitivity

The calculated limit of detection for *P*. *falciparum* was 199.97 parasites /mL blood (Statagraphics Version 18/19). Moreover, even the two low positive samples we missed with the nested PCR, including one *P*. *malariae*, uk3637 (1,000 p/mL) and one *P*. *falciparum*, uk5502 (50 p/mL), were positive in the FRET-qPCR. The results of this study demonstrate that the FRET-qPCR is more sensitive than the “molecular gold standard” (Table 1). Another two low positive samples with 1,000 parasites/mL (uk4605 and uk5848) were not scored by UK NEQAS and declared as educational specimens because less than 50% of the participating laboratories achieved correct results. These two samples gave correct results in both PCRs in this study (Table 1). The results of the 10 fold dilutions of a sample with 1,000,000 *P*. *falciparum*/mL blood (uk5083) revealed an inverse correlation between Cp value and parasite/mL blood, the Cp value rising by 3–4 cycles with every dilution step (Table 2).

**Table 2:** Crossing point (Cp) values of different concentrations of *P*. *falciparum* in the novel FRET-qPCR.

*P*. *falciparum*/ mL	Cp value
1,000,000/ mL	16
100,000/ mL	19
10,000/ mL	23
1,000/ mL	26
100/ mL	29

### Mixed infections

**Fig 3:**
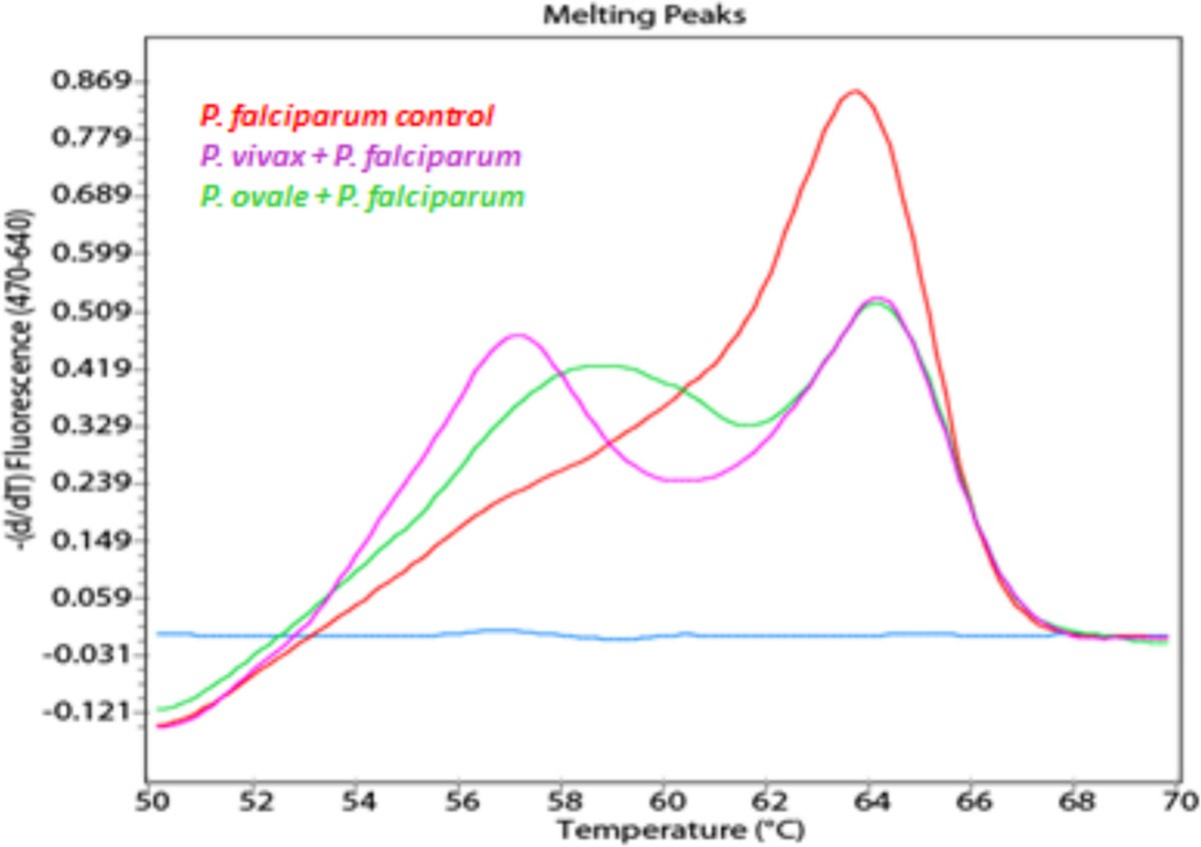
Mixed infections of *P*. *falciparum* with *P*. *vivax* and *P*. *falciparum* with *P*. *ovale*. Melting curves of amplicons post real-time PCR from mixed infections. Red: *P*. *falciparum* positive control; blue: negative control included reaction mixture and water; green: 10,000 *P*. *falciparum*/mL (uk 5083 1:100) + 20,000 *P*. *ovale*/mL (uk 5086); lilac: 10,000 *P*. *falciparum*/mL (uk 5083 1:100) + 6,400 *P*. *vivax*/mL (uk 5365).

No mixed infections were included in the 56 UK NEQAS samples, thus we generated artificial mixed samples of *P*. *falciparum* (10,000 p/mL) with *P*. *vivax* (6,400 p/mL) or *P*. *ovale* (20,000 p/mL), respectively. Both species were successfully differentiated (Fig 3). This experiment was repeated with only 1,000 *P*. *falciparum*/mL and the same concentrations of *P*. *vivax* and *P*. *ovale*, respectively, and the *P*. *falciparum* melting peak was still detected.

## Discussion

In this study, a novel simple and rapid FRET-qPCR detecting all human malaria parasites in a highly sensitive and quantitative way was established and validated with international reference samples. The turn-around time including DNA extraction is less than two hours. The assay can simultaneously differentiate between *P*. *falciparum*, *P*. *vivax*, *P*. *ovale* and *P*. *malariae*, even in mixed infections. *P*. *knowlesi* is detected, but requires an additional PCR for reliable identification. *P*.*ovale curtisi* and *wallikeri* are both detected but cannot be differentiated with our novel assay.

The use of molecular methods for malaria diagnosis in research and clinical care has increased remarkably over the past two decades [15]. They usually have higher sensitivities and specificities compared to microscopy and RDT [8, 16] and are particularly helpful to rule out low-level parasitemia [6, 10, 17]. Of the numerous PCR protocols published, a nested PCR protocol still serves as a “molecular gold standard” [6, 7]. However, it is rather labor-intensive, and the turnaround time is generally too long for routine clinical application [11]. Compared to conventional PCR, real-time PCR has the advantage of faster and quantitative results, the contamination risk is reduced, and it is less labor-intensive [3, 4, 8, 9]. Yin et al. [6] compared 24 different PCR protocols and concluded that it is challenging to select the best protocol for malaria diagnosis.

The novel FRET-qPCR established here, is based on a real-time PCR protocol by Rougemont et al. [8], who reported high sensitivity of 1–10 copies/per reaction for all human *Plasmodium* species. However, up to three Taqman assays (one monoplex followed by two simultaneous multiplex reactions) were necessary to obtain a final species-specific result. Yet, their primers target a conserved region of the 18S rDNA, qualified to design two appropriate FRET probes. The 18S rRNA gene is stable and conserved in all *Plasmodium* species and has 5–7 copies per genome [11, 18]. In combination with the primers Plasmo1 and Plasmo2, the newly designed probes MalFL and MalLC640 allowed both, *Plasmodium* detection and species differentiation, in a single run. The discrimination between *P*. *falciparum*, *P*. *vivax/ knowlesi*, *P*. *ovale*, and *P*. *malariae* is based on the presence of single nucleotide polymorphisms (SNPs) within the sequence targeted by the MalLC640 probe, lowering the Tm in the melting curve analysis from 63.5–66°C for *P*. *falciparum* to 63.0–63.5°C for *P*. *malariae*, 58–60°C for *P*. *ovale*, and 56–57.5°C for *P*. *vivax/knowlesi*, respectively (Fig 1). The ability of our FRET-qPCR to simultaneously detect and differentiate between *Plasmodium* species is an important advantage since treatment is dependent on the infecting species.

It has been argued that only an external quality assurance scheme for the validation of malaria PCR assays in use can ensure accurate diagnosis [6, 15]. Thus, all available 56 reference samples, collected since the validation of molecular malaria diagnostics was introduced by UK NEQAS in 2016, were included in this study. The UK NEQAS reports not only included the *Plasmodium* species, but also the parasite concentration. All UK NEQAS reference samples (56/56) gave correct results in the novel FRET-qPCR, compared to 96.43% correct results in the standard nested PCR performed in our lab from 2016 through 2020 (Table 1). The high specificity of the newly developed test is demonstrated by the fact that all FRET-qPCR positive samples (n = 46) were correctly designated to the reported species based on their Tm values (Table 1, Fig 2). And all negative samples (n = 10) and DNA from *Leishmania*, *Babesia*, *Pneumocystis*, and *Toxoplasma*, organisms which can result in a similar illness, or are biologically similar to malaria parasites, showed no reaction. To evaluate the sensitivity, we produced 10 fold dilutions of one *P*. *falciparum* sample. In general, a 10 fold dilution of the DNA results in approximately three cycles of rising Cp value in real-time PCR. The correlation between the number of parasites/mL and the resulting Cp values was as expected, and down to 100 parasites/mL were detected (Table 2). The calculated LOD of our FRET-qPCR for *P*. *falciparum* was 199.97 parasites /mL blood, equivalent to two copies per FRET-qPCR reaction. Formal external quality assessment programs (EQA) established a threshold of 2,000 parasites/mL to determine “adequate” performance for molecular diagnosis of malaria [15]. Hence, the sensitivity of the novel FRET-qPCR is considerably higher than the claimed threshold and at least 50 fold higher than the sensitivity achieved by an experienced microscopist examining thick and thin blood films [8, 19]. The high sensitivity and specificity of the FRET-qPCR are further demonstrated by achieving 100% correct UK NEQAS results. Down to 50 *P*. *falciparum* parasites/mL blood were detected in one sample (uk5502) and several other samples with low parasitemia (≤1,000 parasites/mL) of *P*. *falciparum* and also other species, were correctly diagnosed (Table 1). High sensitivity is a key issue in the diagnosis of imported malaria, because returning travelers, migrants, and refugees, sometimes taking prophylactic medication or self-medication, often show very low parasitemia. And even if parasitemia is high enough for microscopic detection of *Plasmodium*, species differentiation can be extremely difficult [3, 20]. *P*. *ovale* has been described to be the species most frequently misdiagnosed, for both *P*. *falciparum* and *P*. *vivax*, respectively [4]. Although infections with *P*. *vivax* and *P*. *ovale* are less severe, patients require different treatment regimens to eliminate their chronic liver stages and prevent relapses and would definitely benefit from fast and reliable species-specific diagnosis [19]. Our set of reference samples comprised only samples of *P*. *ovale curtisi*, but we have performed the FRET-qPCR also on 3 routine samples of *P*. *ovale curtisi* and one archived *P*. *ovale wallikeri* DNA sample diagnosed by nested PCR. There was no difference between the Tm values of *P*. *ovale curtisi* and *P*. *ovale wallikeri*.

Just as in microscopy, the detection of mixed infections is challenging also in PCR-based approaches [9, 20]. Here, artificially generated mixed infections of *P*. *falciparum* (10,000 p/mL, and 1,000 p/mL) with *P*. *vivax* (6,400 p/mL) or *P*. *ovale* (20,000 p/mL), respectively, resulted in two melting peaks per sample, allowing precise designation to the correct species (Fig 3).

Because of the smaller difference in melting temperatures between *P*. *falciparum* (Tm 63.5–66°C) and *P*. *malariae* (Tm 63–63.5°C), the detection of mixed infections with these two species would be more difficult and it is possible that *P*. *malariae* in the presence of *P*. *falciparum* might be overlooked. However, *P*. *malariae*, even though the infection can last for many years, is rarely involved in imported malaria [4]. In such cases, besides microscopy, the nested PCR protocol [7] still serves as an excellent tool when it comes to ruling out potential mixed infections of *P*. *falciparum* and *P*. *malariae*. Unfortunately, our method is unable to differentiate between *P*. *vivax* and *P*. *knowlesi*. Until today, we have had no clinical sample of a patient with *P*. *knowlesi* sent in for routine diagnostics, but as this species currently only occurs in a rather well-defined geographical region, the travel history, blood film microscopy and/ or the nested PCR would enable us to differentiate *P*. *knowlesi* from *P*. *vivax* [13, 14]. To our opinion, it is essential to have a simple and rapid assay that detects all species, particularly in travel and migration medicine in non-endemic regions with few malaria cases per year and thus little microscopic experience, but patients coming from all over the world and potentially involving any species. Already Fuehrer et al. [21] recommended PCR-based techniques detecting all human *Plasmodium* species for malaria diagnosis in returning travelers. The determined Cp values, inversely proportional to the initial *Plasmodium* sp. concentration in the sample, were compared with the parasite load/mL reported by UK NEQAS (Table 1). The UK NEQAS samples showed Cp values from 14 to >30, corresponding to 20,000,000–50 parasites/mL. Samples dating from 2016–2018 underwent several freeze-thaw cycles before tested in the FRET-qPCR, which might explain the lower sensitivity in the older samples compared with those from 2019–2020, analyzed in both PCRs within three weeks of receipt. To our observation more than three freeze-thaw cycles were particularly problematic, which has also been reported by others [10, 22].

Apart from first-line diagnostics, the novel FRET-qPCR might serve well in monitoring the response to treatment. A clear correlation of the Cp value with the number of *P*. *falciparum*/mL blood was observed, for example an increase from Cp16 to Cp23 indicates a 10^2^ decrease of parasites/mL (Table 2). The quantification of parasites by real-time PCR is far more convenient and less time-consuming than counting the parasites in stained slides. Rougemont et al. suggested monitoring parasitemia by real-time PCR during the first three days of therapy and described a good correlation with the estimated parasitemia in blood films [8]. A close follow-up is important to detect early treatment failure, especially in *P*. *falciparum* malaria [8, 23].

We have already been performing the novel FRET-qPCR in routine diagnostics on clinical samples for two years now, in parallel to conventional microscopy of Giemsa-stained thick and thin blood films and partly the standard nested PCR. DNA was extracted from 200 μL of EDTA-blood with the same method as described in the Materials and Methods section for UK NEQAS samples. Until now, all FRET-qPCR results of clinical samples were in accordance with conventional diagnostics. We have tested >200 clinical blood samples so far, including samples positive for *P*. *falciparum* (n = 27), *P*. *vivax* (n = 4), *P*. *ovale* (n = 7), and *P*. *malariae* (n = 2), respectively (S1 Table). Until now, we have performed an additional nested PCR only in 3 out of 7 clinical samples positive for *P*. *ovale* in the FRET-qPCR and microscopy, and identified them as *P*. *ovale curtisi;* the other 4 were doubtlessly identified as *P*. *ovale* by microscopy. We are planning to further investigate all *P*. *ovale* FRET-qPCR positive clinical samples by nested PCR in the future because it is not possible to differentiate *P*. *ovale curtisi* from *wallikeri* with the new assay. From the *Plasmodium*-negative clinical samples, 20 were confirmed negative by nested PCR, 115 only by microscopy. Further *Plasmodium* negative whole blood samples came from healthy blood donors without travel histories. For economic reasons we could not perform nested PCR and/or microscopy on all malaria-negative donor blood samples.

In summary, this novel FRET-qPCR allows the simultaneous quantitative and species-specific detection of *Plasmodium* spp. within less than two hours, including DNA extraction. Apart from the obvious advantage as an excellent adjunct and quality control for conventional microscopy, it can also help to monitor treatment outcomes. Moreover, it has the potential to detect asymptomatic infections and might therefore also be helpful in large screenings, including even blood banks screenings in some contexts.

## Supporting information

S1 Fig Melting peaks of amplicons post real-time PCR. Comparison of *P*. *knowlesi* (red), *P*. *vivax* (blue), *P*. *ovale* (grey), *P*. *malariae* (dark green) and 3 different *P*. *falciparum* UK NEQAS samples.
(TIF)

S1 Table Comparison of tests on 224 clinical blood samples. (DOCX)
