# Supplementary figures and images for: Validation of a novel FRET real-time PCR assay for simultaneous quantitative detection and discrimination of human Plasmodium parasites
Source: PLoS One. 2021 Jun 4;16(6):e0252887. doi: 10.1371/journal.pone.0252887 (PMC8177637; doi:10.1371/journal.pone.0252887)

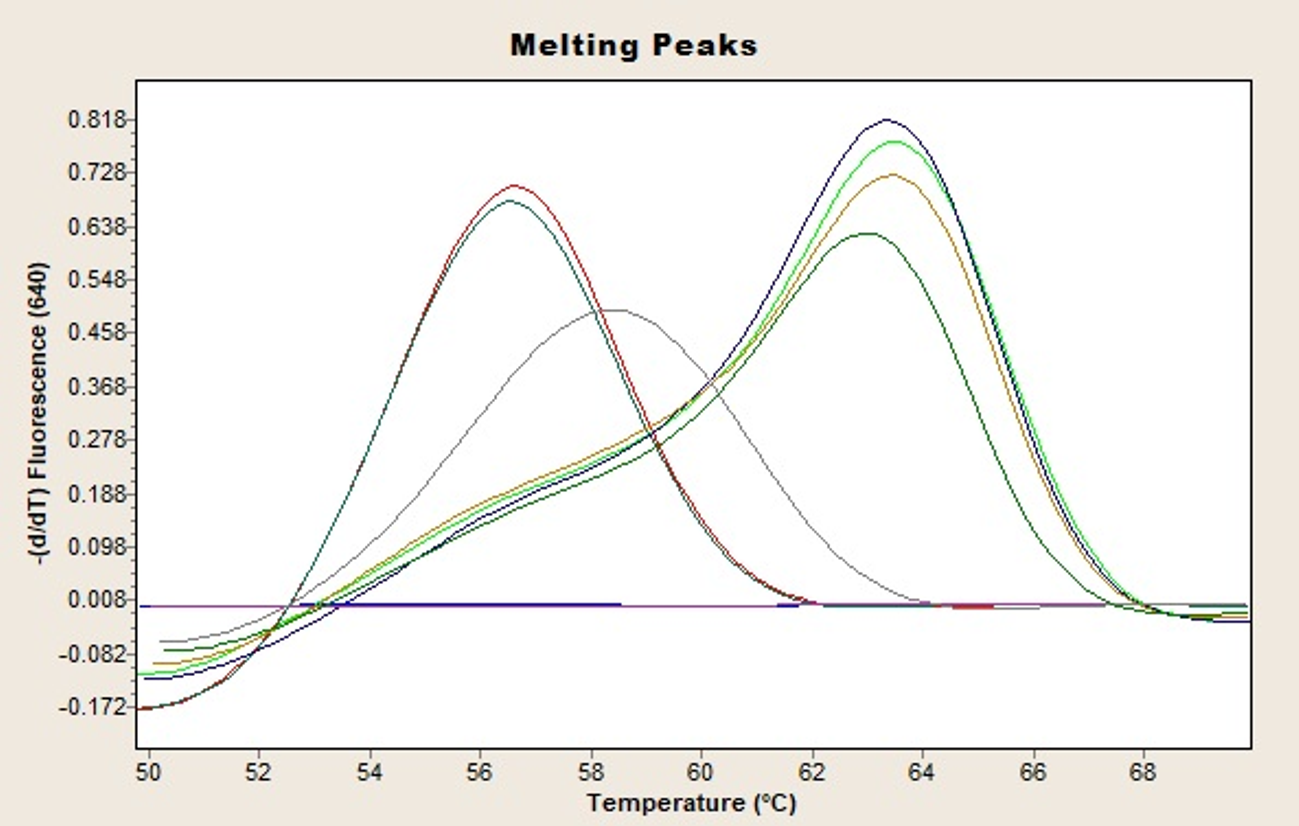

Supplement: S1 Fig — Comparison of P. knowlesi (red), P. vivax (blue), P. ovale (grey), P. malariae (dark green) and 3 different P. falciparum UK NEQAS samples. (TIF) [file pone.0252887.s001.tif]
