# Supplementary material for: Validation of a novel FRET real-time PCR assay for simultaneous quantitative detection and discrimination of human Plasmodium parasites
Source: PLoS One. 2021 Jun 4;16(6):e0252887. doi: 10.1371/journal.pone.0252887 (PMC8177637; doi:10.1371/journal.pone.0252887)
Supplement: S1 Table — (DOCX) [file pone.0252887.s002.docx]

**S1 Table. Comparison of tests on 224 clinical blood samples.**

| ***Plasmodium* species** | **FRET-qPCR results** | **Microscopy results** | **Nested PCR results** |
| --- | --- | --- | --- |
| *P. falciparum* | 27 *P. falciparum* | 25 *P. falciparum*  2 *Plasmodium* sp. | 14 *P. falciparum*  13 nd |
| *P. vivax* | 4 *P. vivax/knowlesi* | 3 *P. vivax*  1 *P. vivax/ovale* | 4 *P. vivax* |
| *P. ovale* | 7 *P. ovale* | 4 *P. ovale*  2 *P. ovale/vivax*  1 *Plasmodium* sp. | 3 *P. ovale curtisi*  4 nd |
| *P. malariae* | 2 *P. malariae* | 2 suspected *P. malariae* | 2 *P. malariae* |
| *Plasmodium* negative patients | 135 negative | 134 negative  1 questionable | 20 negative  115 nd |
| Healthy blood donors | 49 negative | nd | nd |
